# Dysconnectivity of Multiple Brain Networks in Schizophrenia: A Meta-Analysis of Resting-State Functional Connectivity

**DOI:** 10.3389/fpsyt.2019.00482

**Published:** 2019-07-12

**Authors:** Siyi Li, Na Hu, Wenjing Zhang, Bo Tao, Jing Dai, Yao Gong, Youguo Tan, Duanfang Cai, Su Lui

**Affiliations:** ^1^Huaxi MR Research Center (HMRRC), Department of Radiology, West China Hospital, Sichuan University, Chengdu, China; ^2^Department of Radiology, West China Hospital, Sichuan University, Chengdu, China; ^3^Department of Psychoradiology, Chengdu Mental Health Center, Chengdu, China; ^4^Department of Geriatric Psychiatry, The Fourth People’s Hospital of Chengdu, Chengdu, China; ^5^Department of Psychiatry, Zigong Mental Health Center, Zigong, China

**Keywords:** schizophrenia, resting state, magnetic resonance imaging, functional connectivity, brain network, meta-analysis

## Abstract

**Background:** Seed-based studies on resting-state functional connectivity (rsFC) in schizophrenia have shown disrupted connectivity involving a number of brain networks; however, the results have been controversial.

**Methods:** We conducted a meta-analysis based on independent component analysis (ICA) brain templates to evaluate dysconnectivity within resting-state brain networks in patients with schizophrenia. Seventy-six rsFC studies from 70 publications with 2,588 schizophrenia patients and 2,567 healthy controls (HCs) were included in the present meta-analysis. The locations and activation effects of significant intergroup comparisons were extracted and classified based on the ICA templates. Then, multilevel kernel density analysis was used to integrate the results and control bias.

**Results:** Compared with HCs, significant hypoconnectivities were observed between the seed regions and the areas in the auditory network (left insula), core network (right superior temporal cortex), default mode network (right medial prefrontal cortex, and left precuneus and anterior cingulate cortices), self-referential network (right superior temporal cortex), and somatomotor network (right precentral gyrus) in schizophrenia patients. No hyperconnectivity between the seed regions and any other areas within the networks was detected in patients, compared with the connectivity in HCs.

**Conclusions:** Decreased rsFC within the self-referential network and default mode network might play fundamental roles in the malfunction of information processing, while the core network might act as a dysfunctional hub of regulation. Our meta-analysis is consistent with diffuse hypoconnectivities as a dysregulated brain network model of schizophrenia.

## Introduction

Disrupted resting-state functional connectivity (rsFC) involving a number of brain networks has been suggested as the core pathogenesis of schizophrenia (1–3). Most previous studies tried to identify the specific deficits in neural networks related to the disease, which is crucial not only to understand the mechanism of schizophrenia but also to provide potential biomarkers for clinical use. As the most widely used and simply operated analytic technique, seed-based functional connectivity analysis has demonstrated widespread dysconnectivity in schizophrenia, based on the blood oxygen level-dependent (BOLD) time series extracted from regions of interest (ROIs) as seeds (4).

Despite the growing number of seed-based studies on rsFC in patients with schizophrenia (5–8), the results have been controversial regarding either the location of key regions or the effect of dysconnectivity (i.e., increased or decreased connectivity). Taking the default mode network (DMN) for example, some studies have reported decreased rsFC in schizophrenia patients compared with healthy controls (HCs) between the seed ROI at the posterior cingulate cortex (PCC) and other areas within the same network, including the medial prefrontal, lateral parietal, cerebellar, and insular regions (9, 10). In contrast, other researchers have found increased connectivity within the DMN of patients, although the same seed region had been defined (11, 12). Inconsistency might be affected by studied samples and methodologies; however, seed-based studies pave a promising way to reveal the mechanisms of and biomarkers for schizophrenia (13). Therefore, it is necessary to merge divergent findings into a unified model of network functioning for a better characterization of the rsFC patterns. To fill this gap, meta-analysis could be a robust incorporation strategy by classifying seed ROIs and effects within brain networks (14), which has been proven effective to various studies in psychiatric imaging (15, 16). Recently, Dong and colleagues (17) conducted the first meta-analysis to illustrate rsFC within large-scale brain networks in schizophrenia patients. Based on *a priori* templates in which specific seed ROIs were selected before analysis (14, 18–20), they found dominant hypoconnectivities within multiple networks. This study helped to conceptualize schizophrenia in terms of whole-brain rsFC; however, it was inherently constrained by a dependence on *a priori* data and a lack of the simultaneous investigation of brain systems (4, 13, 21).

Alternatively, independent component analysis (ICA) is a common blind source separation approach applied to functional MRI (fMRI) data (22, 23), allowing a fully data-driven exploration of spatiotemporal patterns of synchronized neuronal activity without any predefined seed region (24–26). It creates a reproducible parcellation of functional brain systems, so that bias is reduced while the consideration of data obtained using various atlases is more flexible (25). Using ICA, Mantini and colleagues identified a collection of brain networks based on the correlation between BOLD signals and electroencephalography rhythms (22, 23). These ICA-based networks have previously been used as brain templates in fMRI studies (27, 28), which decomposed the resting-state brain networks into eight spatiotemporal components, as follows: the dorsal attention network (DAN), the central executive network (CEN), the DMN, the core network (CN), the self-referential network (SRN), the somatomotor network (SMN), the visual network (VN), and the auditory network (AN). To our knowledge, no meta-analysis of large-scale rsFC studies in schizophrenia patients has been based on brain network maps using ICA. We aimed to conduct the present meta-analysis to integrate the results of these studies in schizophrenia using the ICA-based brain templates. Our hypothesis was that distributed dysconnectivity would exist within multiple resting-state brain networks in patients with schizophrenia.

## Methods

### Literature Search

Literature searches were performed by four independent reviewers (SLi, NH, JD, and YG) in PubMed (https://www.ncbi.nlm.nih.gov/pubmed) and Embase (https://www.embase.com) for relevant original articles from January 1, 2007, to April 1, 2018. Earlier studies were excluded to reduce potential confounds due to low gradient fields or traditional analysis methods. The keywords included schizophrenia, rest*(-ing), connect* (-ivity), and functional magnetic resonance imaging, or functional MRI, or fMRI, or magnetic resonance imaging or MRI. In addition, the references within each article were carefully checked for further recruitment. Studies were included if they were 1) original MR studies evaluating seed-based rsFC of whole brain or a specified network to compare schizophrenia patients with HCs and 2) studies that provided seed ROI and peak effect coordinates using Montreal Neurological Institute (MNI) or Talairach coordinates. The exclusion criteria were as follows: 1) the field strength of the MR scanner was less than 1.5 T; 2) seed ROI or peak effect coordinates could not be retrieved; 3) entirely identical samples and seed ROIs were reported in distinct articles; or 4) seed ROIs or investigated networks were beyond the abovementioned eight brain-network templates (22, 23). Reports utilizing the same sample but different seed ROIs were labeled as distinct studies. With the exception of one publication comparing combined patients of two groups (schizophrenia with and without hallucinations) with HCs (29), publications in which different subgroups of schizophrenia were each compared with a single HC group were identified as separate studies.

### Data Extraction

The included studies contained the locations and peak effects of significant intergroup differences in rsFC, so the data were extracted and coded by three reviewers (SLi, YT, and DC), as follows, for subsequent meta-analysis. Inconsistencies were resolved by a third assessor (NH).

First, coordinates of each seed ROI and peak effect of each significant intergroup comparison were extracted. If the seed ROI was a spherical area or an anatomical domain from a standard atlas, the region center was calculated to retrieve representative coordinates. For those studies targeting specified networks rather than whole-brain systems, coordinates of each peak location were also collected. All the coordinates and peak effects were converted to the MNI space. Second, we classified the peak coordinates into eight resting-state networks based on previous brain-network templates (22, 23): 1) the DAN, primarily including the left middle and superior occipital gyri, parietal gyrus, inferior and superior parietal gyri, and middle and superior frontal gyri; 2) the CEN, involving the dorsal lateral prefrontal and posterior parietal cortices; 3) the DMN, comprising the PCC/precuneus and the bilateral inferior parietal, middle temporal, angular, superior frontal, and medial frontal gyri; 4) the CN, the key regions of which were the anterior cingulate, bilateral insular cortices, and dorsolateral prefrontal cortex; 5) the SRN, referring to the ventromedial prefrontal and medial orbital prefrontal cortices, gyrus rectus, and pregenual anterior cingulate gyrus; 6) the SMN, relating to the primary sensory-motor cortices, precentral and postcentral gyri, and the supplementary motor area; 7) the VN, encompassing the occipital gyrus and the temporal–occipital regions along with superior parietal gyrus; and 8) the AN, including the bilateral middle and superior temporal gyri, Heschl’s gyrus, and temporal pole (**Supplementary Table 1**). Third, peak effects were classified into hyperconnectivity or hypoconnectivity in the schizophrenia groups based on the effect direction. Hyperconnectivity was defined as increased rsFC (higher positivity or less negativity of connectivity) in schizophrenia patients compared with HCs; hypoconnectivity was defined as decreased rsFC (less positivity or higher negativity of connectivity) in schizophrenia patients compared with rsFC in HCs.

### Multilevel Kernel Density Analysis and *Post Hoc* Tests

To generalize the results of various studies and control publication bias, two reviewers (WZ and BT) conducted multilevel kernel density analysis (MKDA) using the MKDA toolbox (https://canlabweb.colorado.edu/fmri-resources.html) by treating the proportion of studies that were activated in a region rather than the number of peaks as the test statistic (30). In the MKDA procedure, the multilevel nature of the data was taken into account, and then the comparison indicator maps (CIMs) were weighted by study quality and sample size (31). As a consequence, no single CIM could disproportionately influence the meta-analytic results, while more rigorous and larger studies would contribute more to the results (32–35).

First, the peak coordinates of each comparison map from the included studies were separately convolved with a spherical kernel (*r* = 15 mm) to generate the CIMs (30). The CIMs were limited to a maximum value of 1 so that the values across brain voxels were either 1 or 0, which represented a significant effect or no significant effect in the neighborhood, respectively. Second, the proportion of the study comparison maps (activation maps) that indicated hyperconnectivity or hypoconnectivity for each network within a 15-mm radius of each voxel was produced by averaging the CIMs weighted by sample size and a discounting factor (30). Third, differences between the resulting density maps were computed to test for two activated effects: hyperconnectivity and hypoconnectivity. Therefore, activation maps were summarized based on the height mode or extent mode: the height-based activation maps reflected the activation of voxels that exceeded the maximum expected across the whole brain or within specified network by chance only 5% of the time; the extent-based activation maps revealed the activation of clusters of contiguous voxels above the maximum expected in a cluster of that size by chance.

To correct for multiple comparisons, a Monte Carlo simulation was used to establish a familywise error rate threshold of *p* < 0.05 (14, 30). A jack-knife analysis was carried out to test the replicability of the results. We also performed Fisher’s exact tests to assess whether a specific anatomical region accounted for more of a significant effect than other regions from the same network by comparing the differences in effect likelihood ratio (LR) among regions in the same network.

## Results

**Figure 1:**
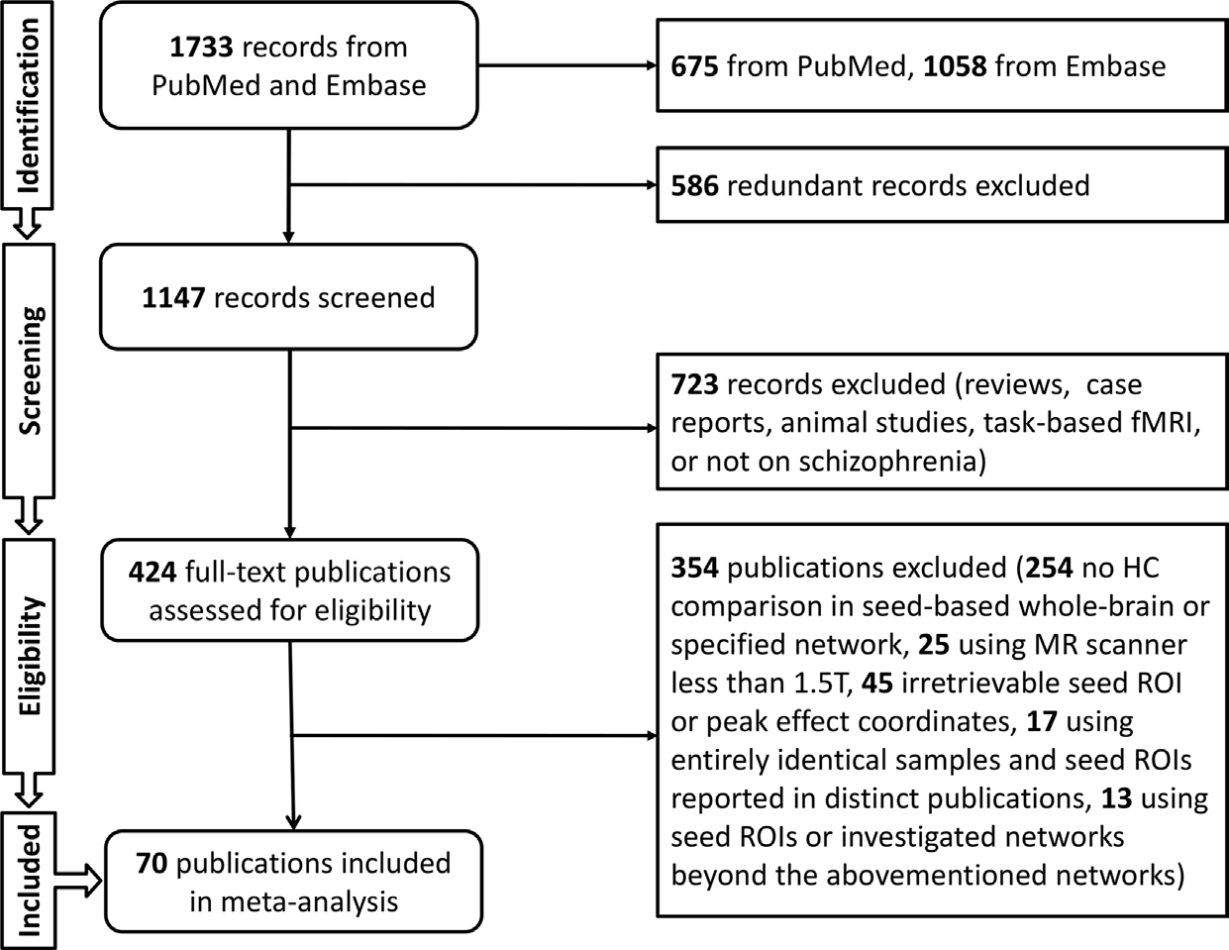
Flowchart of literature search.

The search protocol of meta-analysis followed the Preferred Reporting Items for Systematic Reviews and Meta-Analyses (PRISMA) guidelines (36). The literature searches and criteria yielded a sample of 76 studies from 70 publications (9–12, 29, 37–101) that reported on 2,588 schizophrenia patients and 2,567 HCs. Demographic characteristics were calculated including gender ratio defined as male/female (patients, 1.39; HCs, 1.20), and weighted mean age (patients, 31.00; HCs, 31.17). Weighted mean duration of illness for patients was 89.79 months, and the Positive and Negative Syndrome Scale (PANSS) scores were evaluated (total, 73.46; positive, 18.80; negative, 18.27; general, 34.89). Fifty-five studies provided medication information, and antipsychotic dosages were converted into chlorpromazine equivalents (mean chlorpromazine dosage, 478.58 mg/day) (**Figure 1**, **Supplementary Table 2**).

**Figure 2:**
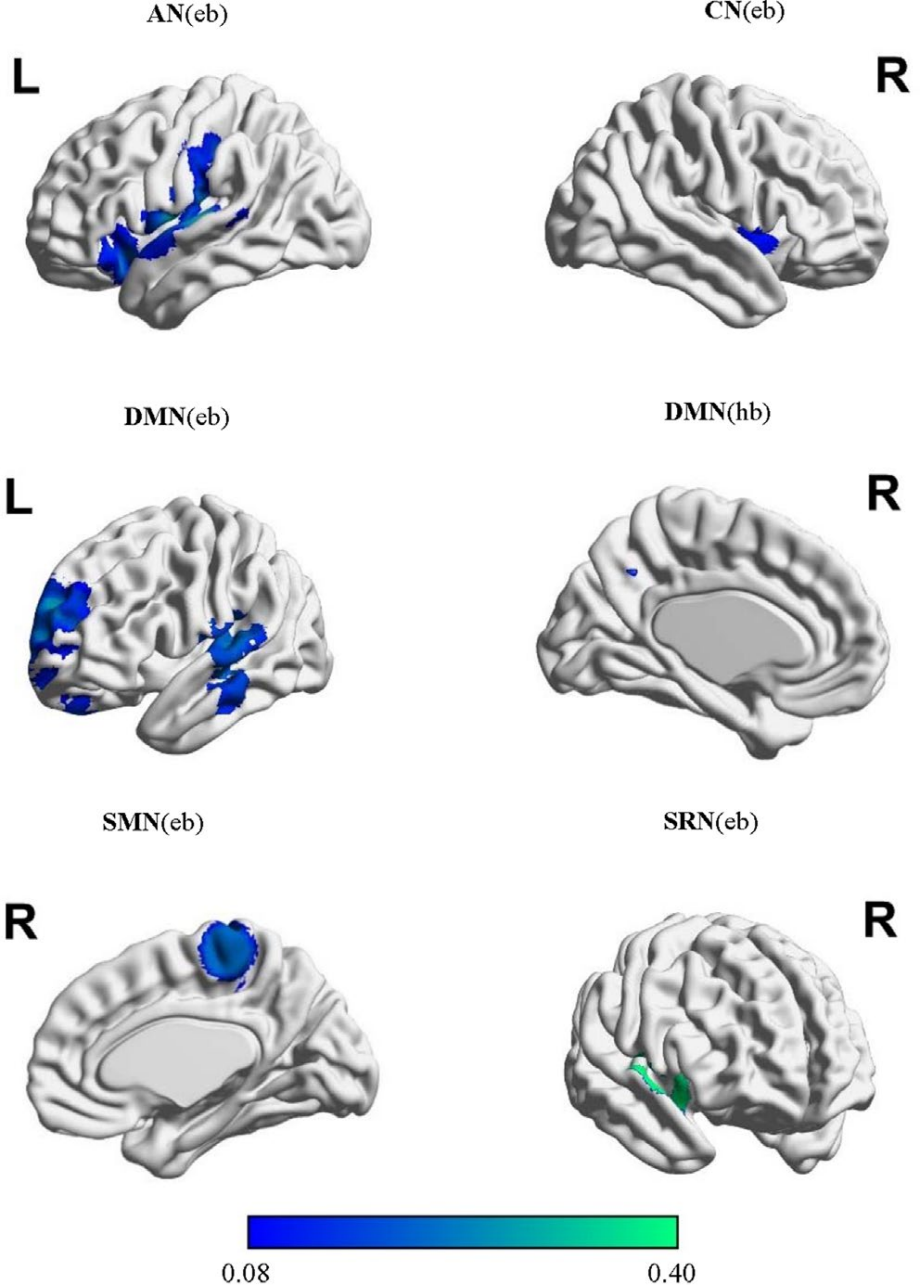
Meta-analysis of abnormal resting-state function connectivity (rsFC) in schizophrenia.

The extracted locations and activation effects of significant intergroup comparisons were classified into the eight networks based on the ICA-based brain templates: the DAN, CEN, DMN, CN, SRN, SMN, VN, and AN (**Supplementary Table 3**). The MKDA showed significant hypoconnectivity between the seed regions and additional areas within the AN, CN, DMN, SRN, and SMN (**Table 1**, **Figure 2**). No hyperconnectivity was detected between the seeds and any other regions within the networks. The jack-knife analysis showed that our results were reliable.

**Table 1:** Results of the meta-analysis of resting-state functional connectivity in patients with schizophrenia.

Seed networks	Effect (threshold)	Effect anatomy	Coordinates			Voxels	Max. *P*	*P*
			x	y	z			
AN	SZ < HC (eb)	Left insula	−42	0	4	7,234	0.29	<0.05
CN	SZ < HC (eb)	Right superior temporal cortex	42	8	−6	1,987	0.22	<0.01
DMN	SZ < HC (hb)	Right medial prefrontal cortex	2	52	2	103	0.26	<0.05
	SZ < HC (eb)	Left precuneus	−2	−68	32	2,282	0.16	<0.01
	SZ < HC (eb)	Left anterior cingulate cortex	0	36	22	5,648	0.17	<0.05
SRN	SZ < HC (eb)	Right superior temporal cortex	48	8	−4	2,463	0.20	<0.01
SMN	SZ < HC (eb)	Right precentral gyrus	50	−6	36	1,682	0.25	<0.05

AN, auditory network; CN, core network; DMN, default mode network; SRN, self-referential network; SMN, somatomotor network; SZ, schizophrenia; HC, healthy control; eb, extent-based; hb, height-based; Max. P, maximum P value.

The coordinates are from the Montreal Neurological Institute space. Voxels represent the sum of 1 × 1 × 1 mm^3^ voxels. Max. P is the maximum statistic of the studies presenting the peak effect weighted mainly by sample size. All the results are significant at p < 0.05 or < .01, corrected for familywise error rate.

In the AN, schizophrenia was associated with decreased rsFC using extent-based threshold between the seed regions and the left insula, which is involved in processing emotional and self-attributed sensory stimuli (102). These seeds included the regions of the bilateral middle and superior temporal gyri, Heschl’s gyrus, and temporal pole. *Post hoc* tests revealed that seeds in the superior temporal gyrus (STG) were more likely than those in the medial prefrontal cortex (MPFC) and frontal pole to exhibit hypoconnectivity with the left insula (LR = 11.76; *p* = 0.003).

In the CN, seed regions were defined as areas within the anterior cingulate, bilateral insular, and dorsolateral prefrontal cortices. Hypoconnectivity was observed in the patients with schizophrenia based on the height threshold between the seeds and the right STG cortex related to the production, interpretation, and self-monitoring of language (103). The likelihood of hypoconnectivity did not differ among anatomical regions within the CN (*p* > 0.05; **Supplementary Table 4**).

In the DMN, seed regions were selected in the PCC/precuneus, bilateral inferior parietal gyrus, angular gyrus, middle temporal gyrus, and superior and medial frontal gyri. The characteristic activated effect in schizophrenia was represented as hypoconnectivity between the seeds and the right MPFC based on the height threshold and the left precuneus and anterior cingulate cortex (ACC) based on the extent threshold. These areas mainly contribute to internal mental participation in self-referential thinking and emotion processing (104–106). *Post hoc* tests indicated that seeds in the ACC, MPFC, and medial cingulate cortex (MCC)/PCC using height-based threshold (LR = 7.11∼10.14; *p* = 0.006∼0.026) and seeds in the MPFC, medial temporal gyrus (MTG)/STG, MCC/PCC, cerebellum, and precuneus on the extent mode (LR = 5.53∼11.23; *p* = 0.003∼0.048) were more likely to exhibit hypoconnectivity either with the right MPFC or with the left ACC and precuneus (**Supplementary Table 4**).

In the SRN, rsFC in patients with schizophrenia was reduced using extent-based threshold between the right STG specialized for self-referential processing (107, 108) and the seeds that included the ventromedial prefrontal cortex, medial orbital prefrontal cortex, gyrus rectus, and pregenual anterior cingulate gyrus. No differences among anatomical seed effects within the SRN were found in *post hoc* tests (*p* > 0.05; **Supplementary Table 4**).

In the SMN, schizophrenia was linked to hypoconnectivity based on the extent threshold between the right precentral gyrus, involved in motor function, and the SMN seeds, including the postcentral and precentral gyri and cerebellum. Compared with the seeds in the cerebellum and Broca’s area, the precentral seeds were more likely to show hypoconnectivity with the right precentral gyrus (LR = 6.90; *p* = 0.041).

## Discussion

The present meta-analysis systematically integrated a sum of 76 studies reporting rsFC of seed-based whole-brain networks and specified networks in patients with schizophrenia based on ICA brain templates, and the meta-analysis consistently indicated that individuals with schizophrenia exhibited large-scale hypoconnectivity involving the auditory system (AN), task and cognitive control (CN), self-referential processing (SRN), episodic memory and self-projection (DMN), and sensory-motor function (SMN). These diffuse disconnections across these brain networks suggest a dysregulated brain network model of schizophrenia in which network dysfunction is associated with deficits in sensory and associated self-referential processing. In this model, decreased coordination within the SRN and DMN might play core roles in the malfunction of information processing, while the CN might act as a dysfunctional hub of regulation.

As a key component of the AN, the STG contains primary auditory cortex and auditory association cortical areas (109, 110). The morphological and functional abnormalities of the STG may be related to the experience of auditory verbal hallucinations (AVHs) and being less responsive to external auditory stimuli during ongoing AVHs compared with when they are absent (111, 112). On the other hand, the SRN is crucial in self-referential processing per se, accounting for differentiating stimuli related to one’s own self from those that are not relevant to inner concerns. The SRN receives information from exteroceptive stimuli through extensive connections from areas associated with primary and secondary auditory sensory modalities (113–118), and it has a role in both bottom-up and top-down modulation between sensory, self-referential, and higher-order processing (119). Furthermore, resting-state networks display a dominant direction of causal influence from the SRN to the AN (28). The dysconnectivity between the SRN seeds and the right STG suggested that the frontotemporal pathways may be a neural substrate underlying dysfunction of auditory and language processing in schizophrenia, i.e., AVHs that are core symptoms of the disease (120, 121). Our findings are in accord with many prior structural and functional neuroimaging studies of hallucinations (121), leading to cognitive models that have implicated disrupted frontotemporal pathways as a potential cause of AVHs and the failure to appropriately monitor inner speech generation.

Decreased rsFC was present in patients with schizophrenia between the DMN seeds and the regions within the right MPFC and left ACC, which is in line with the results of the DMN regarding the within-network connectivity by Dong and colleagues (17). Reduced synchronized neuronal activity within the DMN may support the dysfunctional pattern of schizophrenia in self-related processes relevant to emotional processing, self-referential mental activity, and the recollection of prior experiences (122). The reduced connectivity across the DMN, salience network (SN), and central executive network (CEN) further supports a triple network model in psychopathology (17). Such an imbalanced system of networks fails in dynamic regulation across salience processes, internal mental processes, and goal-relevant external stimuli (123), which is related to multiple phenomenological domains and the total symptom burden of schizophrenia (124, 125). Moreover, our analysis revealed hypoconnectivity between the seeds and the left precuneus, which was beyond the extent of hypoconnectivity described in the DMN by Dong and colleagues (17). This might be due to differences in brain-parcellation modes. It should be noted that the DMN is also thought to be related to AVHs (126, 127), and the anatomical regions of the DMN and SRN partially overlap. However, the SRN exerts a strong directional causal influence over the DMN (28), and the SRN is represented by distinct power spectra of electroencephalography (23) and spatial patterns of the BOLD signal from the DMN (23, 27, 28). Future research designs and phenomenological subcategorizations of individuals with AVHs might facilitate discrimination of different neurocognitive mechanisms (127).

Decreased mutual cross-network effects were exhibited between the AN involving the STG and the CN involving the insula, suggesting weakened synchronization between resting-state networks. Our results are in line with previous structural (128, 129) and functional (130, 131) MR studies in patients with schizophrenia that reported a direct association between the indices of auditory cortex connectivity and psychotic symptoms. Acting as a hub within the CN, the insula is not only connected with the wide array of regions processing external sensory stimuli but also responsible for interoceptive awareness of the body’s internal state, which is uniquely distinct from the external environment. Disrupted interoception processing within the insula would be expected to distort the evaluation of stimuli and boundaries of the self, resulting in allowing internal sensory information to be attributed to an external source (102). Taken together, our results suggest an etiology of AVHs in schizophrenia, leading to dysfunction in distinguishing an internally generated interoception from an external sensory experience (132, 133).

We also detected hypoconnectivity between the SMN seeds and the right precentral gyrus, which implies diminished activity within this network. Compared with HCs, schizophrenia patients showed limited connectivity in areas more strongly related to motor functions, such as the lateral M1 with the supplementary motor and medial motor areas (134). In addition, reduced cortical volume and attenuated activation of the precentral gyrus have been associated with motor-related cognitive dysfunction and impaired behavioral performance in the emotional face task (134–136), which is correlated with the clinical severity of schizophrenia. Although these findings corroborate the dysconnectivity hypothesis of schizophrenia, there are also patterns of increased connectivity within extensive motor networks of the patients (134, 137) and ultrahigh-risk youths (138). Despite the mixed findings, our analysis could be beneficial for better understanding of the relationships across the motor systems.

Further, we analyzed the data with other two organizations of resting state networks (20, 139) to test the repeatability of results (**Supplementary Table 5**). The hypoconnectivity in the DMN is consistent with our results between the seeds and the left precuneus/ACC using Yeo’s and Power’s templates, which makes internal mental participation in self-referential thinking and emotion processing more convincing (122). We also found a similar hypoconnectivity within the SMN in the right precentral gyrus using Yeo’s template, instead of the left postcentral gyrus with Power’s template; changes in different brain regions within the same network may reflect the extensiveness of the dysconnectivity in motor system. Meanwhile, we detected the decreased connectivity across the SMN in schizophrenia in the left ACC both in Yeo’s and Power’s network organizations. This might be due to different network classifications, in which our template included brain regions relating to auditory and visual systems; and functional alternations in the ACC have been found to be associated with attention and sensorimotor processes in patients (140, 141). Using Yeo’s and Power’s templates, we found extensive hypoconnectivities in individually defined networks. The hypoconnectivities between seeds in the limbic network (LN) and left STG/left inferior parietal gyrus were detected using Yeo’s templates, which is consistent with findings in the frontoparietal network (FPN) and DMN in Power’s classification. We also found decreased connectivity in the ventral attention network (VAN) between the seeds and the right middle temporal cortex using Yeo’s template rather than ours and Power’s. In addition to common network definitions including the DMN, SMN, DAN, and FPN, we had some findings in distinctive network definitions using Power’s template. The hypoconnectivities in the left insula within the cingulo-opercular network (CON) and the left parahippocampal gyrus within unknown network (UnN; mostly related to the memory retrieval process) were detected, and the findings in the left insula are consistent with our findings in the AN, while the parahippocampal gyrus participated in the key process of memory (142).

Our study reflected decreased rsFC with affected anatomic regions including left ACC, insula, precuneus and right STG, MPFC, and precentral gyrus. Previous large meta-analyses in cortical and subcortical changes using MRI (143, 144) have revealed structural alternations including widespread thinner cortex and smaller surface area in frontal and temporal regions, as well as reduced volume in the hippocampus, which is mostly consistent with our functional findings in the CN, SRN, and part of the DMN, while changes in precuneus and some subcortical regions are not consistent with our results in other networks. Moreover, we detected different performance of the left and right hemispheres, which was not shown in structural changes. These overlaps and diversities of brain alternations in structure and function existed in different networks and might suggest different pathophysiological process in different networks affected by schizophrenia.

Although our findings support the dysconnectivity hypothesis of schizophrenia (145) and identify regions associated with the pathogenesis of the disease, limitations should be considered in three perspectives. First, the exclusion of negative findings may have biased the results. This should motivate studies using improved methodology beyond MKDA, thereby both positive and negative findings could be matched. Second, the network spatial maps used in our analysis did not give consider the epencephalon, despite its potential role in the cerebello-thalamo-cortical circuit in psychosis (134, 146). A new parcellation template would benefit from more comprehensive network mapping. Third, we did not test whether the results are independent from the different types of schizophrenia patients due to limited number of studies included in the context of each brain network. Analysis with subtypes of schizophrenia would be a strong supplement, and more studies of specific networks will be needed in the future.

In conclusion, the present meta-analysis provides insight into rsFC within multiple brain networks based on ICA templates in schizophrenia and suggests diffuse hypoconnectivities as a dysregulated brain network model of the disease. Future systematic analyses including more homogenous samples are demanded, in view of the relationships across brain networks, and correlations between the levels of connectivity and clinical severity of schizophrenia.

## Data Availability

The datasets generated for this study are available on request to the corresponding author.

## Author Contributions

SLu contributed to the conceptual design and constructive discussion. SLi and NH searched the literature, extracted the data, and wrote the manuscript. WZ and BT performed the MKDA and *post hoc* tests. JD, YG, YT, and DC assisted in data analysis.

## Funding

This study was supported by the National Natural Science Foundation of China (Project Nos. 81671664 and 81621003), National Program for Support of Top-notch Young Professionals (Project No. W02070140), Fundamental Research Funds for the Central Universities (Project No. 2018SCUH0011), Science and Technology Project of the Health Commission of Sichuan Province (Project No. 18ZD035), Sichuan Science and Technology Program (Project No. 2019YJ0155), and 1.3.5 Project for Disciplines of Excellence, West China Hospital, Sichuan University (Project Nos. ZYYC08001 and ZYJC18020).

## Conflict of Interest Statement

The authors declare that the research was conducted in the absence of any commercial or financial relationships that could be construed as a potential conflict of interest.
