# Supplementary material for: Dysconnectivity of Multiple Brain Networks in Schizophrenia: A Meta-Analysis of Resting-State Functional Connectivity
Source: Front Psychiatry. 2019 Jul 12;10:482. doi: 10.3389/fpsyt.2019.00482 (PMC6639431; doi:10.3389/fpsyt.2019.00482)
Supplement: Supplementary file 1 [file DataSheet_1.docx]

# Supplementary Materials

## Supplementary Table 1. Summary of anatomical locations in eight resting-state networks.

| **Classfication of eight resting-state networks** | | | | | | | |
| --- | --- | --- | --- | --- | --- | --- | --- |
|  |  |  |  |  |  |  |  |
| ***Dorsal Attention Network (DAN)*** | | ***Central-Executive Network (CEN)*** | | ***Default Mode Network (DMN)*** | | ***Core Network (CN)*** |  |
| Anatomical Locations | Hem | Anatomical Locations | Hem | Anatomical Locations | Hem | Anatomical Locations | Hem |
| Inferior frontal gyrus, triangular | L | Superior frontal gyrus | L | Superior frontal gyrus, medial | L | Superior frontal gyrus, medial | L |
|  | R |  | R |  | R |  | R |
| Middle frontal gyrus | L | Middle frontal gyrus | L | Precuneus | L | Middle temporal gyrus | L |
|  | R |  | R |  | R |  | R |
| Inferior frontal gyrus, opercular | L | Angular gyrus | L | Superior frontal gyrus | L | Superior temporal gyrus | L |
|  | R |  | R |  | R |  | R |
| Precentral gyrus | L | Precuneus | L | Anterior cingulate gyrus | L | Inferior frontal gyrus, opercular | R |
|  | R |  | R |  | R | Inferior frontal gyrus, triangular | R |
| Inferior parietal gyrus | L | Supramarginal gryus | L | Angular gyrus | L | Anterior cingulate gyrus | L |
| Inferior frontal gyrus, orbital | L |  | R |  | R |  | R |
| Middle occipital gyrus | L | Middle temporal gyrus | R | Posterior cingulate gyrus | L | Median cingulate gyrus | L |
| Postcentral gyrus | L | Postcentral gyrus | L |  | R |  | R |
| Inferior temporal gyrus | L | Middle occipital gyrus | L | Middle frontal gyrus | L | Insula | L |
| Superior parietal gyrus | L |  | R |  | R |  | R |
| Middle temporal gyrus | L | Inferior parietal gyrus | L | Median cingulate gyrus | L |  |  |
|  | R |  | R |  | R |  |  |
| Middle frontal gyrus, orbital | L | Precentral gyrus | L | Inferior frontal gyrus, triangular | L |  |  |
| Supramarginal gryus | L | Middle frontal gyrus, orbital | L |  | R |  |  |
|  |  | Rolandic operculum | L | Inferior parietal gyrus | L |  |  |
|  |  | Inferior frontal gyrus, opercular | L |  | R |  |  |
|  |  | Superior frontal gyrus, medial | R | Superior temporal gyrus, pole | L |  |  |
|  |  | Superior temporal gyrus | L |  |  |  |  |
|  |  |  | R |  |  |  |  |
|  |  |  |  |  |  |  |  |
| ***Self-Referential Network (SRN)*** | | ***Sensory-Motor Network (SMN)*** | | ***Visual Network (VN)*** |  | ***Auditory Network (AN)*** |  |
| Anatomical Locations | Hem | Anatomical Locations | Hem | Anatomical Locations | Hem | Anatomical Locations | Hem |
| Anterior cingulate gyrus | L | Precentral gyrus | L | Calcarine fissure | L | Superior temporal gyrus | L |
|  | R |  | R |  | R |  | R |
| Medial frontal gyrus, orbital | L | Postcentral gyrus | L | Lingual gyrus | L | Heschl gyrus | L |
|  | R |  | R |  | R |  | R |
| Inferior frontal gyrus, orbital | L | Rolandic operculum | L | Middle occipital gyrus | L | Rolandic operculum | L |
|  | R |  | R |  | R |  | R |
| Superior frontal gyrus, orbital | L | Supplementary motor area | L | Cuneus | L | Insula | L |
|  | R |  | R |  | R |  | R |
| Middle frontal gyrus, orbital | L | Superior frontal gyrus, medial | L | Superior occipital gyrus | L | Supramarginal gryus | L |
|  | R | Supramarginal gryus | L |  | R |  | R |
| Superior frontal gyrus, medial | R |  |  | Inferior occipital gyrus | L | Middle temporal gyrus | L |
| Olfactory cortex | L |  |  |  | R |  | R |
|  | R |  |  |  |  | Superior temporal gyrus, pole | R |
| Gyrus rectus | L |  |  |  |  |  |  |
|  | R |  |  |  |  |  |  |

Note: Hemisphere (Hem), left (L), right (R).

## Supplementary Table 2. Summary of demographic and clinical characteristics of studies included in meta-analysis.

| **Reference** | **SCZ**  **(*N*)** | **HC (*N*)** | **Age of SCZ (Mean)** | **Education (y)** | **Field Strength of Scanner** | **Illness Duration ^d^** | **Treated Patients when Studied (%)** | **Mean CPZ-Equivalent （mg/day）** | **PANSS TS**  **(Mean)** | **PANSS PS**  **(Mean)** | **PANSS NS**  **(Mean)** | **PANSS GP**  **(Mean)** |
| --- | --- | --- | --- | --- | --- | --- | --- | --- | --- | --- | --- | --- |
| Anticevic, Tang et al. 2014; Chronic | 20 | 96 | 31.43±8.20 | 11.48±3.52 | 3T | 64.45 m | 95.00 | 240.00 | NA | NA | NA | NA |
| Anticevic, Tang et al. 2014; Early course | 28 | 96 | 25.00±9.70 | 11.54±3.02 | 3T | 4.27 m | 43.00 | 96.40 | NA | NA | NA | NA |
| Anticevic, Hu et al. 2015 | 129 | 106 | 24.29±7.88 | 12.47±3.13 | 3T | 12.62 m | NA | NA | 95.88 | 24.50 | 18.88 | 46.73 |
| Shinn, Baker et al. 2013; Auditory hallucinations | 27 | 28 | 40.00±11.00 | Unk | 3T | 18.00 y | 92.59 | 611.00 | Unk | Unk | Unk | Unk |
| Shinn, Baker et al. 2013; Non-auditory hallucinations | 14 | 28 | 37.00±10.00 | Unk | 3T | 14.00 m | 100.00 | 435.00 | Unk | Unk | Unk | Unk |
| Alonso-Solis, Vives-Gilabert et al. 2015; Auditory verbal hallucinations | 19 | 20 | 40.05±8.90 | Unk | 3T | 16.11 y | 100.00 | Unk | 72.47 | 17.89 | 21.47 | 34.22 |
| Alonso-Solis, Vives-Gilabert et al. 2015; Non-auditory verbal hallucinations | 14 | 20 | 36.43±7.10 | Unk | 3T | 8.00 y | 100.00 | Unk | 55.23 | 11.43 | 14.36 | 27.36 |
| Chai, Whitfield-Gabrieli et al. 2011 | 16 | 15 | 41.60±2.60 | Unk | 3T | Unk | 100.00 | 504.70 | 84.70 | Unk | Unk | Unk |
| Chang, Xi et al. 2015; Auditory verbal hallucinations | 18 | 20 | 22.56± 6.73 | 12.44±2.30 | 3T | 5.94 m | NA | NA | 106.44 | 31.11 | 25.78 | 49.56 |
| Chang, Xi et al. 2015; Non-auditory verbal hallucinations | 18 | 20 | 22.67± 3.85 | 12.56±2.19 | 3T | 12.44 m | NA | NA | 88.06 | 18.61 | 22.06 | 47.39 |
| Chang, Shen et al. 2014 | 25 | 25 | 25.36±6.32 | 12.28±2.57 | 1.5T | 18.32 m | 76.00 | Unk | 77.59 | 18.73 | 21.39 | 37.47 |
| Chen, Tu et al. 2013 | 36 | 36 | 32.90±7.80 | 14.60±1.50 | 3T | 10.00 y | 97.22 | Unk | 60.40 | 14.60 | 15.30 | 30.50 |
| Chen, Liu et al. 2017 | 20 | 20 | 40.30±13.80 | 10.90±2.70 | 3T | Unk | 100.00 | 281.20 | 58.70 | 12.90 | 18.00 | 27.80 |
| Knochel, Stablein et al. 2014 | 21 | 21 | 38.38±10.30 | 15.82±4.92 | 3T | 8.45 y | 100.00 | Unk | 63.20 | 15.40 | 15.11 | 32.60 |
| Mier, Eisenacher et al. 2017 | 22 | 22 | 38.05±9.19 | 10.36±1.65 | 3T | 10.50 y | 100.00 | 452.52 | Unk | 14.32 | Unk | 32.59 |
| Holt, Cassidy et al. 2011 | 18 | 19 | 35.70±13.40 | Unk | 3T | 12.60 y | 83.33 | 329.50 | 49.10 | 12.40 | 12.30 | 24.30 |
| Du, Pearlson et al. 2016 | 82 | 82 | 38.00±14.00 | Unk | 3T | Unk | 100.00 | Unk | Unk | 15.30 | 15.10 | Unk |
| Duan, Gan et al. 2015; Baseline | 68 | 62 | 36.30±7.30 | 12.80±3.40 | 3T | 3.80 y | 94.12 | Unk | 82.30 | 24.60 | 25.30 | 33.60 |
| Duan, Gan et al. 2015; Follow-up | 68 | 62 | 38.50±8.10 | NA | 3T | NA | 86.76 | Unk | 82.30 | 24.60 | 25.30 | 33.60 |
| Duan, Chen et al. 2015 | 28 | 31 | 36.54±11.46 | 11.39±3.08 | 3T | 12.00 y | 100.00 | 329.72 | 63.86 | 16.54 | 19.61 | 27.71 |
| Ganella, Bartholomeusz et al. 2017 | 42 | 42 | 41.30±10.00 | 12.00±0.55 | 3T | 17.90 y | 100.00 | 615.40 | 59.10 | 15.60 | 16.40 | Unk |
| Fan, Tan et al. 2013 | 27 | 15 | 39.70±7.20 | 12.40±2.30 | 3T | 16.50 y | 100.00 | 443.10 | 63.00 | 11.80 | 19.90 | 27.70 |
| Orliac, Naveau et al. 2013 | 26 | 26 | 35.60±8.90 | 11.90±1.70 | 3T | 12.20 y | 100.00 | 326.30 | 50.40 | 12.80 | 12.40 | 25.30 |
| Fang, Wang et al. 2018 | 20 | 22 | 24.20±4.80 | 12.80±3.90 | 3T | 11.60 m | 100.00 | 423.50 | Unk | 23.50 | 19.80 | 42.90 |
| Guo, Tang et al. 2014 | 19 | 19 | 23.95±7.49 | 11.32±2.40 | 3T | 9.37 m | 100.00 | 162.30 | 77.79 | 19.47 | 20.26 | 38.05 |
| Guo, Yao et al. 2014 | 49 | 50 | 22.69±4.62 | 10.94±2.40 | 3T | 22.45 m | NA | NA | 91.31 | 22.27 | 22.82 | Unk |
| Guo, Liu et al. 2017 | 28 | 40 | 22.93±3.92 | 10.54±2.32 | 3T | 24.14 m | NA | NA | 88.11 | 22.68 | 21.18 | Unk |
| Guo, Liu et al. 2017 | 28 | 40 | 22.93±3.92 | 10.54±2.32 | 3T | 24.14 m | NA | NA | 88.11 | 22.68 | 21.18 | 44.25 |
| Guo, Liu et al. 2017 | 28 | 40 | 22.93±3.92 | 10.54±2.32 | 3T | 24.14 m | NA | NA | 88.11 | 22.68 | 21.18 | Unk |
| Yan, Tian et al. 2012 | 30 | 30 | 23.10±3.60 | 13.70±2.00 | 3T | 39.00 m | 100.00 | 407.70 | 67.30 | 19.40 | 16.10 | 31.80 |
| He, Deng et al. 2013 | 115 | 113 | 25.36±8.26 | 12.14±3.06 | 3T | 39.54 w | NA | NA | 92.14 | Unk | Unk | Unk |
| Iwabuchi and Palaniyappan 2017 | 62 | 71 | 38.37±14.2 | 13.19±1.81 | 3T | 16.50 y | Unk | 366.51 | 49.87 | 12.16 | 15.20 | Unk |
| Penner, Ford et al. 2016 | 24 | 24 | 23.20±4.20 | Unk | 3T | 13.70 m | 100.00 | 258.00 | NA | NA | NA | NA |
| Hadley, Nenert et al. 2014 | 21 | 21 | 36.00±10.20 | Unk | 3T | 13.30 y | NA | NA | NA | NA | NA | NA |
| Jiang, Xu et al. 2015 | 20 | 17 | 26.40±8.01 | Unk | 3T | Unk | NA | NA | 81.60 | 20.45 | 15.45 | 39.90 |
| Jiang, Duan et al. 2017 | 20 | 20 | 40.30±13.80 | 10.90±2.70 | 3T | 13.30 y | 100.00 | 281.20 | 58.80 | 12.90 | 18.00 | 27.80 |
| Kirino, Tanaka et al. 2017 | 14 | 15 | 38.10±8.90 | Unk | 3T | 14.30 y | 100.00 | NA | Unk | Unk | Unk | Unk |
| Lang, Wang et al. 2016; Complete remission | 36 | 55 | 32.80±6.20 | Unk | 3T | 117.40 m | 97.22 | 464.80 | 48.60 | 11.60 | 12.90 | 24.20 |
| Lang, Wang et al. 2016; Incomplete remission | 58 | 55 | 34.00±9.60 | Unk | 3T | 126.70 m | 86.21 | 461.00 | 83.50 | 20.50 | 23.60 | 39.40 |
| Moran, Tagamets et al. 2013 | 44 | 44 | 35.20±12.10 | Unk | 3T | Unk | 100.00 | 411.00 | NA | NA | NA | NA |
| Liu, Zhuo et al. 2016 | 95 | 104 | 34.10±9.20 | Unk | 3T | 123.00 m | 90.52 | 452.97 | Unk | 16.83 | 20.20 | 70.04 |
| Liu, Zhang et al. 2018 | 48 | 31 | 15.79±1.64 | 8.88±1.95 | 3T | 5.35 m | NA | NA | 75.10 | 21.50 | 17.92 | 34.25 |
| Lui, Yao et al. 2015 | 37 | 59 | 36.00±14.00 | 14.00±3.00 | 3T | 14.81 y | 86.49 | 483.00 | 71.20 | 18.20 | 17.92 | 34.25 |
| Hoptman, Antonius et al. 2014 | 33 | 31 | 38.20±10.40 | Unk | 3T | 16.50 y | 100.00 | 1112.80 | 74.70 | 19.50 | 19.20 | Unk |
| Hoptman, D'Angelo et al. 2010 | 25 | 21 | 36.70±10.50 | 12.30±2.10 | 3T | Unk | 100.00 | 1157.80 | 78.70 | 18.90 | 20.90 | Unk |
| Cole, Anticevic et al. 2011 | 23 | 22 | 36.54± 9.36 | 13.08±2.10 | 3T | Unk | 100.00 | 589.88 | NA | NA | NA | NA |
| Woodward, Rogers et al. 2011 | 42 | 61 | 36.90±11.90 | 2.90±1.50 | Unk | 15.30 y | 97.62 | Unk | Unk | 19.20 | 13.80 | 32.20 |
| Kraguljac, White et al. 2014 | 22 | 22 | 33.77± 9.27 | Unk | 3T | 9.32 y | NA | NA | NA | NA | NA | NA |
| Mothersill, Tangney et al. 2017 | 27 | 25 | 41.07±10.56 | Unk | 3T | Unk | 77.78 | 356.57 | NA | NA | NA | NA |
| Hoffman, Fernandez et al. 2011; Combined | 32^a^  24^b^ | 23 | 37.20±8.80 ^a^  41.10±10.70 ^b^ | 13.80±2.30 | 3T | Unk | 93.75 ^a^  95.83 ^b^ | 634.00 ^a^  474.00 ^b^ | Unk | 16.50 ^a^  16.60^b^ | 13.70 ^a^  16.10^b^ | 33.0 ^a^  34.80 ^b^ |
| Salvador, Sarro et al. 2010 | 40 | 40 | 41.45±12.90 | Unk | 1.5T | median=20 y, 1^st^ and 3^rd^ quartiles = 11, 24 y | 97.50 | 578 （median） | Unk | Unk | Unk | Unk |
| Salvador, Landin-Romero et al. 2017 | 116 | 122 | 36.76±11.05 | Unk | 1.5T | 14.88 y | 95.69 | 694.27 | 69.23 | 16.57 | 19.57 | 33.08 |
| Berman, Gotts et al. 2016 | 19 | 26 | 19.38±4.87 | Unk | 3T | 9.17 y | 100.00 | Unk | NA | NA | NA | NA |
| Bluhm, Miller et al. 2007 | 17 | 17 | 33.54±13.77 | Unk | 4T | 117.37 m | 88.24 | 231.88 | NA | NA | NA | NA |
| Bluhm, Miller et al. 2009 | 17 | 17 | 33.54±13.77 | Unk | 4T | 117.37 m | 88.24 | 231.88 | NA | NA | NA | NA |
| Schilbach, Hoffstaedter et al. 2016 | 75 | 82 | 33.46±9.61 | Unk | 3T | 9.03 y | 94.70 | Unk | Unk | Unk | Unk | Unk |
| Tian, Meng et al. 2011 | 30 | 30 | 22.63±3.76 | 13.67±1.95 | 3T | 39.00 m | 100.00 | 407.67 | 67.27 | 19.37 | 16.13 | Unk |
| Tu, Hsieh et al. 2012 | 30 | 30 | 30.80±6.53 | 14.60±1.71 | 3T | 8.29 y | 96.67 | Unk | 67.50 | 15.83 | 17.83 | 34.63 |
| Tu, Lee et al. 2013 | 36 | 36 | 32.90±7.80 | 14.60±1.50 | 3T | 10.00 y | 97.22 | Unk | 60.40 | 14.60 | 15.30 | 30.50 |
| Su, Lan et al. 2013 | 25 | 25 | 42.50±9.90 | 11.80±3.50 | 3T | 8.70 y | 100.00 | 263.00 | Unk | 16.60 | 15.20 | 32.60 |
| Oertel-Knochel, Knochel et al. 2013 | 24 | 24 | 37.90±7.84 | 15.08±2.51 | 1.5T | 13.52 y | 100.00 | 610.42 | 63.29 | 15.45 | 15.19 | 32.65 |
| Oertel-Knochel, Knochel et al. 2014 | 24 | 24 | 37.90±7.84 | 15.08±2.51 | 1.5T | 13.52 y | 100.00 | 610.42 | 63.29 | 15.45 | 15.19 | 32.65 |
| Wang, Zhou et al. 2015 | 94 | 102 | 33.60±7.70 | Unk | 3T | 120.10 m | 93.62 | 450.40 | Unk | 16.60 | 20.30 | Unk |
| Wang, Zhang et al. 2018 | 48 | 31 | 15.79±1.64 | 8.88±1.95 | 3T | 5.35 m | NA | NA | 75.10 | 21.50 | 17.92 | 34.25 |
| Wang, Zhan et al. 2018 | 48 | 31 | 15.79±1.64 | 8.88±1.95 | 3T | 5.35 m | NA | NA | 75.10 | 21.50 | 17.92 | 34.25 |
| Wang, Zhang et al. 2017 | 35 | 30 | 15.50±1.80 | 8.50±1.48 | 3T | 16.00 m | NA | NA | 74.67 | 20.42 | 20.91 | 33.28 |
| Wei, Womer et al. 2017 | 45 | 50 | 18.42±3.84 | 10.71±2.43 | 3T | 4.32 m | NA | NA | NA | NA | NA | NA |
| Jung, Jang et al. 2012 | 16 | 23 | 24.75±5.46 | Unk | 1.5T | 4.80 y | 100.00 | Unk | 57.07 | 14.25 | 17.06 | 28.63 |
| Xu, Qin et al. 2015 | 66 | 76 | 33.00±7.60 | Unk | 3T | 114.00 m | 92.42 | 437.40 | Unk | 17.00 | 21.10 | 72.60 |
| Zhang, Shen et al. 2014 | 18 | 18 | 19.70±0.90 | Unk | 3T | Unk | NA | NA | NA | NA | NA | NA |
| Zhang, Zheng et al. 2015 | 37 | 30 | 15.50±1.80 | 8.50±1.48 | 3T | 16.00 m | NA | NA | 74.62 | 20.42 | 20.91 | 33.28 |
| Zheng, Zhang et al. 2016 | 35 | 30 | 15.50±1.76 | 8.70±1.24 | 3T | 6.60 m | NA | NA | 74.62 | 20.42 | 20.91 | 33.28 |
| Zhou, Tan et al. 2010 | 19 | 19 | 15.00~18.00^c^ | Unk | 1.5T | < 6.00 m | NA | NA | Unk | Unk | Unk | Unk |
| Zhou, Liang et al. 2007 | 17 | 17 | 22.90±6.00 | 12.6 ± 2.2 | 1.5T | Unk | 76.47 | Unk | 85.90 | Unk | Unk | Unk |
| Zhou, Shu et al. 2008 | 17 | 14 | 23.70±5.80 | 13.20±2.40 | 1.5T | 44.30 m | 70.59 | 570.80 | 87.60 | 21.40 | 21.50 | Unk |
| Zhou, Ma et al. 2015 | 91 | 100 | 33.80±7.70 | Unk | 3T | 120.10 m | Unk | 447.40 | Unk | 16.60 | 20.00 | Unk |
|  | Sum=  2588 | Sum=  2567 | M^e^=31.00 | M^f^=11.70 |  | M^f^=89.79m |  | M^F^=478.58 | M^f^=73.46 | M^f^=18.80 | M^f^=18.27 | M^f^=34.89 |

Abbreviations: SCZ, schizophrenia patients; HC, healthy control; CPZ, chlorpromazine; PANSS, Positive and Negative Symptom Scale; TS, total score; PS, positive score; NS, negative score; GP, general psychopathology score; Unk, unknown; NA, not applicable.

^a^ Hallucinating patients;

^b^ Nonhallucinating patients;

^c^ Range of age (years);

^d^ Illness duration could be reported by years (y), months (m), or weeks (w) depending on original studies.

^e^ Sample size weighted mean value;

^f^ Sample size weighted mean value (only available for subsamples)

## Supplementary Table 3. Summary of networks and seed-ROIs of studies included in meta-analysis.

| **Reference** | **Network and Activated Effect** | | | | | | | | | | | | | | | |
| --- | --- | --- | --- | --- | --- | --- | --- | --- | --- | --- | --- | --- | --- | --- | --- | --- |
|  | DAN | | CEN | | DMN | | CN | | SRN | | SMN | | VN | | AN | |
|  | SCZ  >HC | SCZ  <HC | SCZ  >HC | SCZ  <HC | SCZ  >HC | SCZ  <HC | SCZ  >HC | SCZ  <HC | SCZ  >HC | SCZ  <HC | SCZ  >HC | SCZ  <HC | SCZ  >HC | SCZ  <HC | SCZ  >HC | SCZ  <HC |
| Anticevic, Tang et al. 2014; Chronic |  |  |  |  |  |  |  |  |  |  |  |  |  |  |  | amyg |
| Anticevic, Tang et al. 2014; Early course |  |  |  |  |  |  |  |  |  |  |  |  |  |  |  | amyg |
| Anticevic, Hu et al. 2015 | MPFC, LPFC | MPFC, LPFC |  |  |  |  | LPFC | LPFC | MPFC | MPFC |  |  |  |  |  |  |
| Shinn, Baker et al. 2013; Auditory hallucinations |  |  |  |  |  |  |  |  |  |  |  |  |  |  | STG | STG |
| Shinn, Baker et al. 2013; Non-auditory hallucinations |  |  |  |  |  |  |  |  |  |  |  |  |  |  |  | STG |
| Alonso-Solis, Vives-Gilabert et al. 2015; Auditory verbal hallucinations |  |  |  |  | MPFC, PCC, IPL, RSP, TP | MPFC, ParaHIPP, IPL, TP |  |  |  |  |  |  |  |  | MPFC | MPFC |
| Alonso-Solis, Vives-Gilabert et al. 2015; Non-auditory verbal hallucinations |  |  |  |  | IPL, RSP | TP |  |  |  |  |  |  |  |  |  |  |
| Chai, Whitfield-Gabrieli et al. 2011 |  |  |  |  | MPFC |  |  |  |  |  |  |  |  |  |  |  |
| Chang, Xi et al. 2015; Auditory verbal hallucinations |  |  |  |  |  | ACC, SPL |  | ACC |  | ACC |  |  |  |  |  |  |
| Chang, Xi et al. 2015; Non-auditory verbal hallucinations |  |  |  |  |  |  |  |  |  |  |  | PRCG |  |  |  | STG |
| Chang, Shen et al. 2014 | IFG | IPL, SFG |  |  | MTG |  |  |  |  |  |  |  |  |  |  |  |
| Chen, Tu et al. 2013 | cereb | cereb |  |  | cereb | cereb |  | cereb | cereb | cereb | cereb | cereb |  |  |  |  |
| Chen, Liu et al. 2017 |  |  |  |  |  |  |  | ACC |  | ACC |  |  |  |  |  |  |
| Knochel, Stablein et al. 2014 |  |  |  |  | HIPP | HIPP |  |  |  |  |  |  |  |  |  |  |
| Mier, Eisenacher et al. 2017 |  |  |  |  |  |  |  |  |  |  |  |  |  |  |  | STG |
| Holt, Cassidy et al. 2011 |  |  |  |  | MCC/PCC | MCC/PCC |  |  |  |  |  |  |  |  |  |  |
| Du, Pearlson et al. 2016 |  |  |  |  |  | DMPFC, MTG |  |  |  |  |  |  |  |  |  |  |
| Duan, Gan et al. 2015; Baseline |  |  |  |  | HIPP |  |  |  |  |  |  |  |  |  |  |  |
| Duan, Gan et al. 2015; Follow-up |  |  |  |  | HIPP | HIPP |  |  |  |  |  |  |  |  |  |  |
| Duan, Chen et al. 2015 |  |  |  |  | caud | caud |  |  |  |  |  |  |  |  |  |  |
| Ganella, Bartholomeusz et al. 2017 |  |  |  |  |  | PrC, PACG |  |  |  |  |  |  |  |  |  | STG |
| Fan, Tan et al. 2013 |  |  |  |  | MPFC | MPFC |  |  | MPFC | MPFC |  |  |  |  | MPFC | MPFC |
| Orliac, Naveau et al. 2013 |  |  |  |  |  | PCC |  |  |  |  |  |  |  |  |  |  |
| Fang, Wang et al. 2018 |  |  |  | DLPFC |  |  |  |  |  |  |  |  |  |  |  |  |
| Guo, Tang et al. 2014 |  |  |  |  | PCC | MPFC, MTG |  |  |  |  |  |  |  |  |  | STG |
| Guo, Yao et al. 2014 |  |  |  |  | PCC | MPFC, MPG |  |  |  |  |  |  |  |  |  |  |
| Guo, Liu et al. 2017 |  |  |  |  |  | PrC |  |  |  |  |  |  |  |  |  |  |
| Guo, Liu et al. 2017 |  |  |  |  | PCC, MPFC | STG, SPL, cereb |  |  |  |  |  |  |  |  |  |  |
| Guo, Liu et al. 2017 |  |  |  |  | PCC, MPFC |  |  |  |  |  |  |  |  |  |  |  |
| Yan, Tian et al. 2012 |  |  |  |  |  |  | ACC | ACC | ACC | ACC |  |  |  |  |  |  |
| He, Deng et al. 2013 |  |  |  |  |  | PCC |  |  |  |  |  |  |  |  |  |  |
| Iwabuchi and Palaniyappan 2017 |  |  |  |  |  | PrC, thalam |  |  |  |  |  |  |  |  |  |  |
| Penner, Ford et al. 2016 |  | PFC |  |  |  | PFC |  | Insular |  |  |  |  |  |  |  |  |
| Hadley, Nenert et al. 2014 |  |  |  |  |  | VTA |  |  |  |  |  |  |  |  |  |  |
| Jiang, Xu et al. 2015 |  |  |  |  |  | MPFC |  |  |  |  |  |  |  |  |  |  |
| Jiang, Duan et al. 2017 |  |  | DLPFC | DLPFC | PCC | PCC | DAI | DAI |  |  |  |  |  |  |  |  |
| Kirino, Tanaka et al. 2017 |  |  |  |  | PrC | PrC |  |  |  |  |  |  |  |  |  |  |
| Lang, Wang et al. 2016; Complete remission |  |  |  |  |  |  |  |  |  |  |  | PRCG |  | IOG, FG |  |  |
| Lang, Wang et al. 2016; Incomplete remission |  |  |  |  |  |  |  |  |  |  |  | PRCG |  | IOG, FG |  |  |
| Moran, Tagamets et al. 2013 |  |  |  |  |  | PCC |  | DLPFC |  |  |  |  |  |  |  |  |
| Liu, Zhuo et al. 2016 | IPL |  |  |  |  |  |  |  |  |  |  |  |  |  |  |  |
| Liu, Zhang et al. 2018 |  |  |  |  |  | STG, MFG, PrC |  |  |  |  |  | PRCG, POCG, PACG |  |  |  |  |
| Lui, Yao et al. 2015 |  |  |  |  |  |  |  |  | ACC | ACC | PRCG, POCG |  |  |  |  |  |
| Hoptman, Antonius et al. 2014 |  |  |  |  | ACC | ACC | LOPFC | LOPFC | MOPFC | MOPFC |  |  |  |  |  |  |
| Hoptman, D'Angelo et al. 2010 |  |  |  |  |  | MPFC |  |  |  |  |  |  |  |  |  | amyg |
| Cole, Anticevic et al. 2011 |  |  |  |  | PFC | PFC | DLPFC | DLPFC |  |  |  |  |  |  |  |  |
| Woodward, Rogers et al. 2011 |  | IPS, SPL |  | DLPFC | PCC |  |  |  |  |  |  |  |  |  |  |  |
| Kraguljac, White et al. 2014 |  |  |  |  |  | HIPP |  |  |  |  |  |  |  |  |  |  |
| Mothersill, Tangney et al. 2017 |  |  |  |  | PrC, ACC | PrC | ACC |  | ACC |  |  |  |  | TPJ |  |  |
| Hoffman, Fernandez et al. 2011 |  |  |  |  |  |  |  |  |  |  |  |  |  |  | STG | STG |
| Salvador, Sarro et al. 2010 |  |  |  |  | MPFC |  |  |  |  |  |  |  |  |  |  |  |
| Salvador, Landin-Romero et al. 2017 |  |  |  |  | PCC | PCC | LPFC | LPFC |  |  | PRCG | PRCG |  | OG |  |  |
| Berman, Gotts et al. 2016 |  | MFG, SFG |  |  |  | IPL |  |  |  |  |  |  |  |  |  | STG |
| Bluhm, Miller et al. 2007 |  |  |  |  |  | PCC |  |  |  |  |  |  |  |  |  |  |
| Bluhm, Miller et al. 2009 |  |  |  |  |  | RSP |  |  |  |  |  |  |  |  |  |  |
| Schilbach, Hoffstaedter et al. 2016 |  |  |  |  |  | PrC, PCC |  |  |  |  |  |  |  |  |  |  |
| Tian, Meng et al. 2011 |  |  |  |  |  |  |  |  |  |  |  |  |  |  | amyg | amyg |
| Tu, Hsieh et al. 2012 |  |  |  |  |  | ACC |  |  |  |  |  |  |  |  |  |  |
| Tu, Lee et al. 2013 |  | IPL |  | LPFC |  | MPFC, LPFC |  | LPFC | MCC | MCC |  |  |  |  |  | FP |
| Su, Lan et al. 2013 |  |  |  |  |  | DLPFC |  | DLPFC |  |  |  |  |  |  |  |  |
| Oertel-Knochel, Knochel et al. 2013 |  |  |  |  | STG | STG |  |  |  |  |  |  | STG | STG | STG | STG |
| Oertel-Knochel, Knochel et al. 2014 |  |  |  |  |  |  |  |  |  |  |  |  |  |  |  | STG |
| Wang, Zhou et al. 2015 |  |  |  |  | ACC, MCC, PCC | ACC, MCC, PCC |  |  |  |  |  |  |  |  |  |  |
| Wang, Zhang et al. 2018 | MPFC | IPL |  |  | MPFC | IPL |  |  |  |  |  | PRCG |  |  |  | STG |
| Wang, Zhan et al. 2018 |  |  |  |  | MPFC | MPFC |  |  |  | MPFC |  |  |  |  |  |  |
| Wang, Zhang et al. 2017 |  |  |  |  | PrC | PrC |  |  |  |  |  |  |  |  |  |  |
| Wei, Womer et al. 2017 |  |  |  |  |  |  |  | DLPFC |  |  |  |  |  |  |  | amyg |
| Jung, Jang et al. 2012 |  |  |  |  |  |  |  |  |  |  |  | Broca’s area |  |  |  |  |
| Xu, Qin et al. 2015 |  |  |  |  |  | PCC, STG |  |  |  |  |  |  |  |  |  | STG |
| Zhang, Shen et al. 2014 |  |  |  |  | PCC, IPL, PFC, PrC | PCC, IPL, PFC, PrC |  |  |  |  |  |  |  |  |  |  |
| Zhang, Zheng et al. 2015 |  |  |  |  |  | STG, MTG |  |  |  |  |  |  |  | MTG |  |  |
| Zheng, Zhang et al. 2016 |  |  |  |  |  | PrC |  |  | OFC |  |  |  |  |  |  |  |
| Zhou, Tan et al. 2010 |  |  |  |  |  | HIPP, MTG, PrC |  |  |  |  |  |  |  |  |  |  |
| Zhou, Liang et al. 2007 |  |  | DLPFC | DLPFC |  |  | DLPFC | DLPFC |  |  |  |  |  |  |  |  |
| Zhou, Shu et al. 2008 |  |  |  |  | HIPP | HIPP |  |  |  |  |  |  |  |  |  |  |
| Zhou, Ma et al. 2015 |  | FP |  |  |  | FP |  |  |  |  |  |  |  |  |  | FP |

Note: The peak coordinates were classified into eight resting-state networks including the dorsal attention network (DAN), the central executive network (CEN), the default mode network (DMN), the core network (CN), the self-referential network (SRN), the somatomotor network (SMN), the visual network (VN), and the auditory network (AN). The anatomical regions where the seed-ROIs located included hippocampus (HIPP), ventral tegmental area (VTA), medial prefrontal cortex (MPFC), lateral prefrontal cortex (LPFC), dorsolateral prefrontal cortex (DLPFC), dorsal anterior insula (DAI), medial orbital prefrontal cortex (MOPFC), lateral orbital prefrontal cortex (LOPFC), dorsomedial prefrontal cortex (DMPFC), temporo-parietal junction (TPJ), retrosplenial cortex (RSP), frontal pole (FP), temporal pole (TP), inferior parietal lobule (IPL), amygdala (amyg), anterior cingulate cortex (ACC), medial cingulate cortex (MCC), posterior cingulate cortex (PCC), caudate (caud), cerebellum (cereb), medial temporal gyrus (MTG), superior temporal gyrus (STG), medial frontal gyrus (MFG), superior frontal gyrus (SFG), superior parietal lobule (SPL), precuneus (PrC), precentral gyrus (PRCG), postcentral gyrus (POCG), paracentral gyrus (PACG), occipital gyrus (OG), inferior occipital gyrus (IOG), fusiform gyrus (FG), intraparietal sulcus (IPS), ventral tegmental area (VTA), thalamus (thalam) and parahippocampus (ParaHIPP). SCZ, schizophrenia patients; HC, healthy control.

## Supplementary Table 4. Post hoc tests moderated by seed-ROI locations.

| **Seed-network & Threshold** | **Seed Anatomy** | **Effect Network** | **Effect Anatomy** | **Moderated by Anatomy** | **Likelihood Ratio** | ***P*-value** |
| --- | --- | --- | --- | --- | --- | --- |
| AN | amyg, STG, MPFC, FP |  |  |  |  |  |
| SCZ<HC (eb) |  | AN, CN | Left insula | amyg vs STG | 4.956 | 0.058 |
|  |  |  |  | amyg vs MPFC | 1.586 | 0.410 |
|  |  |  |  | amyg vs FP | 1.586 | 0.410 |
|  |  |  |  | STG vs MPFC | 11.756 | 0.003^*^ |
|  |  |  |  | STG vs FP | 11.756 | 0.003^*^ |
|  |  |  |  | MPFC vs FP | 0.000 | 1.000 |
| CN | LPFC, DLPFC, ACC, insula, cereb |  |  |  |  |  |
| SCZ<HC (eb) |  | AN, CN | Right superior temporal cortex | LPFC vs DLPFC | 0.142 | 1.000 |
|  |  |  |  | LPFC vs ACC | 0.172 | 1.000 |
|  |  |  |  | LPFC vs insula | 0.791 | 0.663 |
|  |  |  |  | LPFC vs cereb | 2.171 | 0.343 |
|  |  |  |  | DLPFC vs ACC | 0.623 | 0.697 |
|  |  |  |  | DLPFC vs insula | 1.586 | 0.410 |
|  |  |  |  | DLPFC vs cereb | 3.356 | 0.184 |
|  |  |  |  | ACC vs insula | 0.228 | 1.000 |
|  |  |  |  | ACC vs cereb | 1.152 | 0.606 |
|  |  |  |  | insula vs cereb | 0.365 | 1.000 |
| DMN | ACC, MPFC, MTG/STG, HIPP, MCC/PCC, cereb, PrC, SPL/IPL, caud/thalam |  |  |  |  |  |
| SCZ<HC (hb) |  | DMN, SRN, AN, DAN | Right medial prefrontal cortex | ACC vs MPFC | 10.144 | 0.006^*^ |
|  |  |  |  | ACC vs MTG/STG | 5.730 | 0.053 |
|  |  |  |  | ACC vs HIPP | 2.085 | 0.354 |
|  |  |  |  | ACC vs MCC/PCC | 7.125 | 0.026^*^ |
|  |  |  |  | ACC vs cereb | 0.357 | 1.000 |
|  |  |  |  | ACC vs PrC | 1.115 | 0.612 |
|  |  |  |  | ACC vs SPL/IPL | 1.115 | 0.612 |
|  |  |  |  | ACC vs caud/thalam | 0.000 | 1.000 |
|  |  |  |  | MPFC vs MTG/STG | 0.732 | 0.570 |
|  |  |  |  | MPFC vs HIPP | 3.409 | 0.127 |
|  |  |  |  | MPFC vs MCC/PCC | 0.314 | 0.780 |
|  |  |  |  | MPFC vs cereb | 7.108 | 0.022^*^ |
|  |  |  |  | MPFC vs PrC | 4.980 | 0.059 |
|  |  |  |  | MPFC vs SPL/IPL | 4.980 | 0.059 |
|  |  |  |  | MPFC vs caud/thalam | 10.144 | 0.006^*^ |
|  |  |  |  | MTG/STG vs HIPP | 1.005 | 0.508 |
|  |  |  |  | MTG/STG vs MCC/PCC | 0.088 | 1.000 |
|  |  |  |  | MTG/STG vs cereb | 3.414 | 0.147 |
|  |  |  |  | MTG/STG vs PrC | 1.954 | 0.301 |
|  |  |  |  | MTG/STG vs SPL/IPL | 1.954 | 0.301 |
|  |  |  |  | MTG/STG vs caud/thalam | 5.730 | 0.053 |
|  |  |  |  | HIPP vs MCC/PCC | 1.680 | 0.335 |
|  |  |  |  | HIPP vs cereb | 0.751 | 0.671 |
|  |  |  |  | HIPP vs PrC | 0.162 | 1.000 |
|  |  |  |  | HIPP vs SPL/IPL | 0.162 | 1.000 |
|  |  |  |  | HIPP vs caud/thalam | 2.085 | 0.354 |
|  |  |  |  | MCC/PCC vs cereb | 4.549 | 0.081 |
|  |  |  |  | MCC/PCC vs PrC | 2.849 | 0.182 |
|  |  |  |  | MCC/PCC vs SPL/IPL | 2.849 | 0.182 |
|  |  |  |  | MCC/PCC vs caud/thalam | 7.125 | 0.026^*^ |
|  |  |  |  | cereb vs PrC | 0.219 | 1.000 |
|  |  |  |  | cereb vs SPL/IPL | 0.219 | 1.000 |
|  |  |  |  | cereb vs caud/thalam | 0.357 | 1.000 |
|  |  |  |  | PrC vs SPL/IPL | 0.000 | 1.000 |
|  |  |  |  | PrC vs caud/thalam | 1.115 | 0.612 |
|  |  |  |  | SPL/IPL vs caud/thalam | 1.115 | 0.612 |
| SCZ<HC (eb) |  | DMN, CN, SRN, SMN | Left precuneus | ACC vs MPFC | 2.347 | 0.225 |
|  |  |  | Left anterior cingulate cortex | ACC vs MTG/STG | 2.347 | 0.225 |
|  |  |  |  | ACC vs HIPP | 0.460 | 0.737 |
|  |  |  |  | ACC vs MCC/PCC | 3.203 | 0.139 |
|  |  |  |  | ACC vs cereb | 0.734 | 0.675 |
|  |  |  |  | ACC vs PrC | 4.152 | 0.083 |
|  |  |  |  | ACC vs SPL/IPL | 0.125 | 1.000 |
|  |  |  |  | ACC vs caud/thalam | 2.047 | 0.359 |
|  |  |  |  | MPFC vs MTG/STG | 0.000 | 1.000 |
|  |  |  |  | MPFC vs HIPP | 0.743 | 0.568 |
|  |  |  |  | MPFC vs MCC/PCC | 0.069 | 1.000 |
|  |  |  |  | MPFC vs cereb | 5.530 | 0.048^*^ |
|  |  |  |  | MPFC vs PrC | 0.267 | 0.797 |
|  |  |  |  | MPFC vs SPL/IPL | 1.402 | 0.378 |
|  |  |  |  | MPFC vs caud/thalam | 8.278 | 0.014^*^ |
|  |  |  |  | MTG/STG vs HIPP | 0.743 | 0.568 |
|  |  |  |  | MTG/STG vs MCC/PCC | 0.069 | 1.000 |
|  |  |  |  | MTG/STG vs cereb | 5.530 | 0.048^*^ |
|  |  |  |  | MTG/STG vs PrC | 0.267 | 0.797 |
|  |  |  |  | MTG/STG vs SPL/IPL | 1.402 | 0.378 |
|  |  |  |  | MTG/STG vs caud/thalam | 8.278 | 0.014^*^ |
|  |  |  |  | HIPP vs MCC/PCC | 1.261 | 0.402 |
|  |  |  |  | HIPP vs cereb | 2.315 | 0.263 |
|  |  |  |  | HIPP vs PrC | 1.890 | 0.274 |
|  |  |  |  | HIPP vs SPL/IPL | 0.106 | 1.000 |
|  |  |  |  | HIPP vs caud/thalam | 4.305 | 0.108 |
|  |  |  |  | MCC/PCC vs cereb | 6.765 | 0.025* |
|  |  |  |  | MCC/PCC vs PrC | 0.065 | 1.000 |
|  |  |  |  | MCC/PCC vs SPL/IPL | 2.084 | 0.252 |
|  |  |  |  | MCC/PCC vs caud/thalam | 9.725 | 0.007* |
|  |  |  |  | cereb vs PrC | 8.072 | 0.013* |
|  |  |  |  | cereb vs SPL/IPL | 1.452 | 0.432 |
|  |  |  |  | cereb vs caud/thalam | 0.353 | 1.000 |
|  |  |  |  | PrC vs SPL/IPL | 2.869 | 0.161 |
|  |  |  |  | PrC vs caud/thalam | 11.227 | 0.003* |
|  |  |  |  | SPL/IPL vs caud/thalam | 3.127 | 0.201 |
| SRN | MPFC, ACC, cereb, MCC |  |  |  |  |  |
| SCZ<HC (eb) |  | AN | Right superior temporal cortex | MPFC vs ACC | 0.000 | 1.000 |
|  |  |  |  | MPFC vs cereb | 2.532 | 0.303 |
|  |  |  |  | MPFC vs MCC | 2.532 | 0.303 |
|  |  |  |  | ACC vc cereb | 2.532 | 0.303 |
|  |  |  |  | ACC vs MCC | 2.532 | 0.303 |
|  |  |  |  | cereb vs MCC | 0.000 | 1.000 |
| SMN | PRCG,cereb,Broca's area |  |  |  |  |  |
| SCZ<HC (eb) |  | SMN, DMN | Right precentral gyrus | PRCG vs cereb | 6.904 | 0.041* |
|  |  |  |  | PRCG vs Broca's area | 6.904 | 0.041* |
|  |  |  |  | cereb vs Broca's area | 0.000 | 1.000 |

Note: Likelihood ratio for each pairwise comparison, and *p-*value were reported moderated by anatomy. The anatomical regions where the seed-ROIs located included amygdala (amyg), superior temporal gyrus (STG), medial prefrontal cortex (MPFC), frontal pole (FP), lateral prefrontal cortex (LPFC), dorsolateral prefrontal cortex (DLPFC), anterior cingulate cortex (ACC), insula, cerebellum (cereb), medial temporal gyrus (MTG)/STG, hippocampus (HIPP), medial cingulate cortex (MCC)/ posterior cingulate cortex (PCC), MCC, precuneus (PrC), superior parietal lobule (SPL)/ inferior parietal lobule (IPL), caudate (caud)/thalamus (thalam), precentral gyrus (PRCG) and Broca’s area. The effect networks which exhibited within-network hypoconnectivity included the auditory network (AN), the core network (CN), the default mode network (DMN), the self-referential network (SRN), and the somatomotor network (SMN). eb, extent-based threshold; hb, height-based threshold.

## Supplementary Table 5. Results of the meta-analysis of resting-state functional connectivity in patients with schizophrenia using Yeo and Power’s templates.

1. Results of the meta-analysis of resting-state functional connectivity in patients with schizophrenia using Yeo’s template.

| Seed Networks | Effect (Threshold) | Effect Anatomy | Coordinates | | | Voxels | Max. *P* | *P* |
| --- | --- | --- | --- | --- | --- | --- | --- | --- |
|  |  |  | x | y | z |  |  |  |
| LN | SZ<HC (hb) | Left superior temporal cortex | -56 | -44 | 20 | 26 | 0.44 | <0.001 |
|  |  | Left inferior parietal gyrus | -62 | -46 | 22 | 12 | 0.44 | <0.001 |
| VAN | SZ<HC (eb) | Right middle temporal cortex | 50 | -4 | -26 | 632 | 0.13 | <0.05 |
| DMN | SZ<HC (hb) | Right medial frontal cortex | 2 | 44 | -14 | 3 | 0.24 | <0.001 |
|  |  | Left anterior cingulate cortex | -4 | 50 | 0 | 300 | 0.29 | <0.001 |
|  |  | Left precuneus | -2 | -54 | 36 | 18 | 0.23 | <0.001 |
|  | SZ<HC (eb) | Left anterior cingulate cortex | -1 | 34 | 20 | 5975 | 0.17 | <0.05 |
| SMN | SZ<HC (eb) | Right precentral gyrus | 14 | -28 | 68 | 790 | 0.22 | <0.001 |
|  |  | Left anterior cingulate cortex | -8 | 48 | 10 | 2173 | 0.23 | <0.01 |

1. Results of the meta-analysis of resting-state functional connectivity in patients with schizophrenia using Power’s template.

| Seed Networks | Effect (Threshold) | Effect Anatomy | Coordinates | | | Voxels | Max. *P* | *P* |
| --- | --- | --- | --- | --- | --- | --- | --- | --- |
|  |  |  | x | y | z |  |  |  |
| FPN | SZ<HC (hb) | Right angular cortex | 34 | -52 | 38 | 24 | 0.35 | <0.001 |
|  | SZ<HC (eb) | Right inferior parietal gyrus | 36 | -40 | 44 | 1099 | 0.29 | <0.001 |
| CON | SZ<HC (eb) | Left insula | -38 | 8 | 2 | 810 | 0.26 | <0.001 |
| DMN | SZ<HC (hb) | Left precuneus | -4 | -56 | 38 | 19 | 0.26 | <0.001 |
|  | SZ<HC (eb) | Left superior temporal cortex | -44 | -24 | 12 | 11 | 0.13 | <0.01 |
|  | SZ<HC (eb) | Left precuneus | -8 | -52 | 12 | 6040 | 0.16 | <0.05 |
| SMN | SZ<HC (hb) | Left postcentral gyrus | -54 | -16 | 16 | 12 | 0.25 | <0.001 |
|  | SZ<HC (eb) | Left anterior cingulate cortex | -8 | 48 | 10 | 1827 | 0.19 | <0.01 |
| UnN | SZ<HC (eb) | Left parahippocampa gyrus | -26 | -32 | -12 | 1122 | 0.34 | <0.001 |
